# Association between Negative Life Events and Somatic Symptoms: A Mediation Model through Self-Esteem and Depression

**DOI:** 10.3390/bs13030243

**Published:** 2023-03-10

**Authors:** Sijia Lv, Tong Chang, Siyu Na, Lei Lu, Erying Zhao

**Affiliations:** 1 Harbin Medical University Cancer Hospital, Harbin Medical University, Harbin 150081, China; 2 Psychological Science and Health Management Center, Harbin Medical University, Harbin 150081, China; 3 Department of Engineering Science, University of Oxford, Oxford OX1 3PJ, UK; 4 Department of Psychiatry, University of Oxford, Oxford OX3 7JX, UK

**Keywords:** somatic symptoms, negative life events, self-esteem, depression, serial multiple mediation

## Abstract

The purpose of this study was to investigate the serial multiple mediation of self-esteem and depression in the relationship between negative life events and somatic symptoms in Chinese medical students. We recruited a total of 3383 medical students for this study, and used the Patient Health Questionnaire-15 to assess the somatic symptoms of subjects; the Adolescent Self-Rating Life Events Check List to assess negative life events; the Rosenberg Self-Esteem Scale to assess self-esteem; and the Patient Health Questionnaire for depression. Descriptive analysis and statistical tests were then performed on the collected data. We showed that 39.17% of the medical students had mild somatic symptoms, 24.14% had moderate somatic symptoms, and 5.66% had severe somatic symptoms; we observed significant differences in somatic symptoms among genders, living expenses, and one-child. For negative life events, interpersonal stress was the most important predictor of somatization during the regression analysis. In addition, we observed significance for both the direct and part of the indirect paths from negative life events to somatic symptoms using mediation model analysis. However, we noted that there was no significance for the path through negative life events and self-esteem to somatic symptoms. This study revealed a high prevalence of somatic symptoms among Chinese medical students, and the findings suggested that interventions aimed at reducing somatization in this population should consider the impact of negative life events, particularly those related to interpersonal stress. One potential approach to mitigating the effects of negative life events on somatization is to enhance self-esteem and decrease the level of depression among medical students.

## 1. Introduction

Somatic symptom disorder is characterized by an extreme focus of a person on physical symptoms, such as pain, headaches, weakness, or shortness of breath, which causes significant distress and/or impairment in daily functioning [1]. These symptoms are commonly observed among adolescents [2,3]; for example, a previous study found that the prevalence of somatic symptoms was 22.7% among 15–16-year-old adolescents in a cross-sectional study in Sweden [4]. Although somatic symptoms are typically not life-threatening, they can affect individuals’ daily lives, such as sleep, psychological well-being, and social activities, which can reduce their quality of life and interfere with learning and work abilities. In addition, somatic symptoms often result in repeated and prolonged medical treatments, which can impose a significant economic burden on society [5,6].

The pathogenesis of somatic symptoms is unclear, i.e., medically unexplained symptoms (MUSs) [7]. However, previous studies have suggested that negative life events may play a significant role in the development and course of somatic symptoms [8]. Negative life events are often described as sudden and dramatic experiences, which have the potential to significantly alter an individual’s social world (e.g., unemployment, discrimination, or financial hardship) [9,10]. Proposed mechanisms for the association between negative life events and somatic symptoms involve physiological and emotional stress responses [8]. These negative events often elicit unpleasant emotional experiences, such as unease, helplessness, negativity, anxiety, and so on, which can lead to the development of negative emotional states in individuals. For example, children or adolescents in their early stages of psychological development are particularly susceptible to the impact of negative life events and are at a higher risk of developing somatic symptoms [11]. Additionally, previous research showed that the college students who have experienced high levels of childhood trauma reported more somatic symptoms, likely due to the stress associated with the transition period from adolescence to adulthood [12]. In particular, the college students face a range of academic and social demands and need to adapt to various psychological changes in preparation for their working lives, which can make this period one of the most stressful periods of an individual’s life.

In addition to the impact of negative life events, research suggests that somatic symptoms are closely associated with depression [13,14]. A study found positive correlations between depression and somatic symptoms, including headache, stomach ache, constipation, diarrhea, backache, and fatigue [14]. A cross-cultural study confirmed this link in both Japanese and American undergraduates, with significant correlations found between depression and somatic symptoms for both men and women [15]. Notably, depression and depressive symptoms are prevalent among medical students, with a rate of 27.2% among 122,356 individuals across 43 countries [16]. Studies have suggested that Chinese medical students with a mean prevalence of 32.74% in particular suffer from more severe depression [17].

Negative life events can be significant stressors that contribute to the development of depressive symptoms in adolescents [18]. Prolonged exposure to such events can lead to feelings of hopelessness and helplessness, which may, in turn, result in the development of depressive symptoms. Common stressors experienced by college students often pertain to school life, such as academic failure, and interpersonal relationships, including conflicts with roommates. Medical students, in particular, may face higher levels of stress due to the length of their schooling and clinical practice. These acute stressors can significantly disrupt mental well-being and potentially lead to the emergence of depressive episodes [19]. Maladaptive emotion regulation strategies mediate the relationship between relational stress and challenging life events and depressive symptoms [20]. On the basis of these factors, we propose our first hypothesis that depression may serve as an intermediary variable between negative life events faced by medical students and somatic symptoms.

To investigate the relationship between negative life events and somatic symptoms, we propose another potential pathway that is mediated by self-esteem. This is because self-esteem is a crucial aspect of an individual’s self-concept, which involves their overall assessment of their positive or negative value [21]. People with low self-esteem often have a negative self-evaluation, leading to reduced confidence and courage in dealing with life’s challenges [22]. Previous research has explored the mediating role of self-esteem and its impact on social life in different adolescent groups, including left-behind children [23], college students [24], and medical students [25]. Negative life events can cause a decline in self-esteem, as reported in previous studies, while higher self-esteem has been associated with better social adaptation and increased peer trust and perceived social support [26,27]. Self-esteem has been shown to influence individual cognition, motivation, emotion, and behavior [27]. Therefore, we propose our second hypothesis that self-esteem may mediate the relationship between negative life events faced by medical students and somatic symptoms.

According to the susceptibility model of depression, negative self-evaluation is considered a risk factor for depression [28]. Since self-esteem is a core component of an individual’s self-evaluation, it may also have a negative predictive effect on depression. Previous research found that higher self-esteem can decrease the likelihood of an individual experiencing depression [29]. Negative life events may cause low self-esteem [26], which may exacerbate or even trigger depression, ultimately leading to somatic symptoms. Therefore, self-esteem and depression may have a chain relationship as mediators for negative life events leading to somatic symptoms.

The multiple mediation model is an approach that involves two or more mediation variables between independent and dependent variables. It has been widely used for analyzing the relationships in mental health [30] and social behavior [31]. This model has been shown promising performance in exploring the underlying mechanisms of the relationship between the independent and dependent variables. However, there are limited studies that have examined the association between negative life events and somatic symptoms while considering the self-esteem and depression of medical students, particularly among Chinese medical students. Therefore, this study investigates the serial multiple mediation of self-esteem and depression in the relationship between negative life events and somatic symptoms, with the aim of revealing how negative life events, as an external social factor, may act on somatization through internal cognitive and emotional psychological factors. Ultimately, this study will provide evidence for potential interventions that could alleviate somatic symptoms experienced by medical students.

## 2. Methods

### 2.1. Study Design and Participants

A cross-sectional study was conducted to collect data for this research at Harbin Medical University, China. A total of 3383 clinical medical students were invited to participate in the experiment. Participants were first informed about the objective of the study, and they were assured that no personal identities would be disseminated. Each subject was then asked to complete a voluntary and anonymous questionnaire, which included sociodemographic information, such as age, gender, living expenses, and whether the subject was the only child in their family (i.e., one-child). Four standard scales were used to assess somatic symptoms, negative life events, self-esteem, and depression for each participant. The details of these scales can be found in the following sections. After distributing the scales to all 3383 students, we collected the responses and excluded incomplete scales, resulting in 3219 effective responses, with a response rate of 95.16%. Of the participants with effective scales, 1399 were male (43.46%) and 1820 were female (56.54%), with an average age of 20.92 ± 0.94 years old. The study was approved by the Ethics Committee of Harbin Medical University.

### 2.2. Measurement of Somatic Symptoms

The Patient Health Questionnaire-15 (PHQ-15) was used to assess the somatic symptoms of the subjects in this study [32]. This self-rating scale is a suitable tool to measure the severity of somatization disorders and somatic symptoms. The PHQ-15 consists of 15 items distributed in three dimensions, including cardiothoracic, gastrointestinal, and general discomfort. Each item is rated on a 3-point scale (0 = not bothered at all, 1 = bothered a little, 2 = bothered a lot). The total score ranges from 0 to 30, and higher scores indicate greater severity of somatic symptoms. Somatic symptom severity is categorized into four levels: non or minimal (0–4), low level (5–9), medium level (10–14), and high level (≥15).

### 2.3. Measurement of Negative Life Events

The Adolescent Self-Rating Life Events Check List (ASLEC) was used to assess the negative life events experienced by the participants [33]. This questionnaire was designed to evaluate the occurrence and impact of negative life events in five dimensions, including interpersonal stress (such as being misunderstood or blamed by others), academic pressure (such as having a heavy academic burden), punishment (such as criticism or corporal punishment), personal loss (such as the death of friends or relatives), and adaptation (such as a deterioration in living habits). The scale consists of 27 items, and participants were asked to report whether the listed events had occurred in the past 12 months and to rate the degree of their impact using a 6-point scale ranging from 0 (never experienced) to 5 (experienced and very strongly impacted). A higher score indicates more negative life events experienced. The ASLEC has previously been shown to have good reliability (Cronbach’s α = 0.91) and validity in the Chinese population [34].

### 2.4. Measurement of Self-Esteem

The situation of self-esteem of subjects was assessed by the Self-Esteem Scale (SES) [35]. This questionnaire is a commonly used self-report tool for evaluating an individual’s self-esteem. Participants were presented withasked to answer 10 items, such as “*I feel that I have a number of good qualities*”, and all items in SES were rated on a 4-point scale ranging from “*strongly agree*” to “*strongly disagree*”. Five of the items, such as “*I feel I do not have much to be proud of*”, were reverse scored. The average score was then calculated and used as a measure of self-esteem, with higher scores indicating higher levels of self-esteem.

### 2.5. Measurement of Depression

The depression of subjects was assessed by the Patient Health Questionnaire (PHQ-9). The PHQ-9 is a widely used screening, diagnostic, and monitoring tool for measuring the severity of depression, as recommended by the American Psychiatric Association [1]. Participants are asked to evaluate the presence of 9 symptoms over the previous two-week period, including depressed mood, anhedonia, sleep problems, fatigue, changes in appetite or weight, feelings of guilt or worthlessness, difficulty concentrating, feelings of sluggishness or agitation, and suicidal ideation. Responses are rated on a 4-point scale ranging from 0 (not at all) to 3 (nearly every day). The total score can range from 0 to 27, with higher scores indicating greater severity of depression. Severity is categorized into five levels: none-minimal (0–4), mild (5–9), moderate (10–14), moderately severe (15–19), and severe (20–27).

### 2.6. Statistical Methods

Descriptive analysis was used to describe the summary of the demographic characteristics of the participants. The independent samples *t*-test and one-way analysis of variance (ANOVA) were utilized to compare demographic variables, such as gender, living expenses, and one-child, as well as the distribution of somatic symptoms. Spearman correlation analysis was performed to examine the relationships between four variables: negative life events, self-esteem, depression, and somatic symptoms. Statistical software SPSS version 24.0 was used to conduct these analyses. To evaluate the significance of the multiple mediator model, we employed Model 6 of the PROCESS macro for SPSS. This method is based on ordinary least-squares regression and the bootstrap method. The 1000 bootstrap bias-corrected 95% confidence intervals (BC cIs) were used for mediation analyses in the test from the Serial Multiple Mediation Model 6. Statistical significance was defined as *p* < 0.05.

## 3. Results

### 3.1. Somatic Symptoms of Medical Students

The statistical results of somatic symptoms experienced by medical students are presented in Table 1. Out of the 3219 medical students, only 999 (31.03%) did not suffer somatic symptoms, 1261 (39.17%) reported mild somatic symptoms, 777 (24.14%) had moderate somatic symptoms, and 182 (5.66%) had severe somatic symptoms. There are 15 types of somatic symptom selections in the questionnaire PHQ-15, and Table 2 presents the statistics of the medical students with somatic symptoms. The numbers of people suffering these symptoms from the most to the least are constipation, loose bowels, or diarrhea, 1875 (84.46%); feeling tired or having low energy, 1819 (81.94%); nausea, gas, or indigestion, 1732 (78.02%); headaches, 1637 (73.74%); stomach pain, 1440 (64.86%); feeling heart pound or race, 1417 (63.83%); back pain, 1306 (58.83%); pain in arms, legs, or joints, 1289 (58.06%); trouble sleeping, 1276 (57.48%); menstrual cramps with periods, 1210 (54.5%); chest pain, 899 (40.5%); dizziness, 899 (40.5%); shortness of breath, 672 (30.27%); fainting spells, 208 (9.37%); and pain or problems during sexual intercourse, 172 (7.75%).

### 3.2. Statistical Analysis

The statistical test results of somatic symptoms in different groups are presented in Table 3. The table shows that there are significant differences in somatic symptoms between genders (*p* < 0.05), with females having a significantly higher degree of somatic symptoms (8.37 ± 4.52) than males (5.44 ± 4.15). The ANOVA results reveal significant differences in somatic symptoms among medical students with different living expenses and one-child (*p* = 0.005). In particular, medical students who had higher living expenses or were not the only child in the family experienced more severe somatic symptoms. However, no significant differences were found in somatic symptoms among different emotional statuses and religious beliefs.

### 3.3. Correlation Analysis

The results of the correlation analysis between negative life events, self-esteem, depression, and somatic symptoms are presented in Table 4. As shown in the table, there were positive relationships between negative life events and self-esteem, depression, and somatic symptoms. Furthermore, self-esteem was positively correlated with depression and somatic symptoms, and depression was positively related to somatic symptoms. These correlation analysis findings provide the necessary basis for the subsequent mediating effect test.

### 3.4. Regression Analysis

We conducted regression analysis to further examine the predictive effect of different types of negative life events on the somatization scores. As shown in Table 5, the regression results indicate that punishment, loss, interpersonal relationship pressure, and learning pressure were positively associated with somatization. In particular, interpersonal stress had the most significant predictive effect on somatization. However, adaptive life events showed negative predictive effects on somatization.

### 3.5. Mediation Analysis

**Figure 1:**
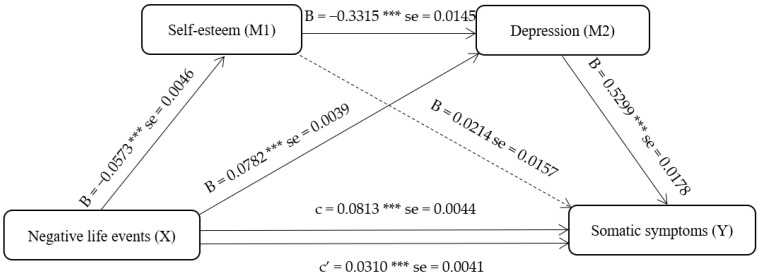
Serial multiple mediation of self-esteem and depression in the relationships between negative life events and somatic symptoms with non-standardized beta values and standard errors. Note: *** *p* < 0.001.

We established a mediating model with negative life events as the independent variable, somatic symptoms as the dependent variable, and self-esteem and depression as mediating variables. As shown in Figure 1, the results of the mediating effect test indicate that negative life events of adolescents not only had direct effects on somatic symptoms but also had indirect effects on somatic symptoms through self-esteem and depression. The significant pathways include negative life events → somatic symptoms; negative life events → depression → somatic symptoms; and negative life events → self-esteem → depression → somatic symptoms. All mediation models were partial mediation models.

The Bootstrap method was used to determine the serial multiple mediation of self-esteem and depression between the four variables. As shown in Table 6, the results of the mediation test demonstrate that the following paths were statistically significant: the path through negative life events to somatic symptoms (point estimate = 0.0310; 95% BC CI [0.0229, 0.0391]), the path through negative life events → self-esteem → depression → somatic symptoms (point estimate = 0.0101; 95% BC CI [0.0079, 0.0125]), and the path through negative life events → depression → somatic symptoms (point estimate = 0.0414; 95% BC CI [0.0363, 0.0467]). However, the path through negative life events → self-esteem → somatic symptoms (point estimate = −0.0012; 95% BC CI [−0.0030, 0.0006]) was observed as not significant. The results indicate that self-esteem and depression may play a sequential mediating role between negative life events and somatic symptoms. Therefore, this model does not conform to the characteristics of the composite multiple mediation model.

## 4. Discussion

Somatic symptoms have received considerable attention among adolescents in recent years. Our study is the first to examine whether depression and self-esteem can moderate the relationship between negative life events and somatic symptoms among medical students in the north of China. The study yielded three important findings: First, the prevalence of somatic symptoms was high among medical students in China, and a significant correlation was found among negative life events, self-esteem, depression, and somatic symptoms. Second, different types of negative life events had varying predictive effects on somatic symptoms, with negative life events about interpersonal pressure having the most significant influence. Third, self-esteem and depression played roles to mediate negative life events and somatic symptoms, but only partially.

The study found that 68.97% of medical students experience somatic symptoms, underscoring the need for greater attention to the prevention and intervention of somatization in this population. The most commonly reported symptoms were general discomfort (including headache, dizziness, fatigue, and insomnia), consistent with previous findings [36]. The questionnaire PHQ-15 involved 15 typical somatic symptoms and revealed that constipation, loose bowels, or diarrhea; feeling tired or having low energy; and nausea, gas, or indigestion were the top three symptoms reported. These symptoms may be attributed to the prolonged years of study, heavier academic burden, and the high-level requirements of medical education that medical students face in comparison to their peers. Additionally, medical students are a vulnerable population, as they must manage multiple stressors, which may contribute to the development of somatic distress [37].

This study found that female medical students scored higher than male students in somatic symptom problems, which is consistent with the results in previous studies [38,39]. This gender difference may be attributed to females being more sensitive, vulnerable, and influenced by their surroundings, leading to a deeper perception of negative life events. Moreover, medical students who were not the only child faced more severe somatic symptoms, potentially due to the lack of parental support. Being the only one child in the family, the student may enjoy more resources and less life pressure, which can reduce the likelihood of developing somatic symptoms. Interestingly, medical students with higher living expenses experienced more severe somatic symptoms. However, the relationship between living expenses and somatic symptoms remains unclear, and further research is needed to explore potential influences, such as life experiences, eating habits, and school environment.

This study demonstrates a direct relationship between negative life events and somatic symptoms in medical students. The greater the number of negative life events experienced by medical students, the higher the likelihood of somatic symptoms. Additionally, the results indicate a significant correlation between depression and self-esteem with negative life events and somatic symptoms. Medical students with higher levels of depression reported more negative life events, more somatic symptoms, and lower self-esteem, while those with lower self-esteem showed more negative life events, somatic symptoms, and depression. These findings align with previous research; for example, a study on suicidality in Chinese medical students found a positive correlation between adverse life events, depressive symptoms, and somatic symptoms [40]. Other studies have also demonstrated that individuals who grow up in environments with poor family harmony and support may be more susceptible to somatic disorders [41,42]. Furthermore, different levels of self-esteem are strongly associated with the development of depression, with self-esteem considered a risk factor for depression in young adults [43]. Therefore, the correlations between negative life events, self-esteem, depression, and somatic symptoms are supported by extensive evidence.

To further examine the relationship between negative life events and somatic symptoms, we conducted regression analysis to test the predictive effect of different types of negative life events on somatic scores. The results showed that punishment, personal loss, interpersonal relationship pressure, and learning pressure had a positive predictive effect on somatization, while adaptive life events had a negative predictive effect. Of these, interpersonal stress had the most significant predictive effect on somatization. This finding is supported by previous research demonstrating that interpersonal factors are important predictors of somatic symptoms in the general population [44]. Previous research found that interpersonal stress was significantly related to both psychological distress and somatic symptoms, both directly and indirectly through pathways mediated by loneliness [45]. It is acknowledged that experiencing discrimination can be detrimental to one’s relationships, and research has shown that even ethnic microaggressions can lead to an increase in depression and somatic symptoms in adolescents [46]. As college students mature, they become increasingly aware of the importance of interpersonal relationships, and the influence of interpersonal relationship pressure becomes more powerful. It is interesting to note that negative life events classified as academic stress were found to have the least predictive effect on somatic symptoms among medical students. It is understood that college students with many years of learning experience are more strongly influenced by psychological and social factors, such as interpersonal relationships, personal loss, punishment, and social adaptation. These results suggest the need to pay more attention to the different types of negative experiences that medical students face to decrease the level of somatic symptoms.

The primary objective of this study was to examine the moderating role of depression and self-esteem in the relationship between negative life events and somatic symptoms. By utilizing the Bootstrap method, we analyzed the chain-mediating model, and the results suggest that self-esteem and depression may play sequential and mediating roles between negative life events and somatic symptoms. All paths in the model were significant, with the exception of the association between self-esteem and somatic symptoms. Negative life events were found to have a direct effect on somatic symptoms and can be modulated by depression alone. Moreover, the sequence of negative events was found to first affect self-esteem, then depression, and, finally, somatic symptoms, as demonstrated by the chain of events.

Negative life events have been found to have a direct effect on depression, which is consistent with previous research demonstrating that negative life events can increase depression levels [47]. Prior studies have also investigated the relationship between life events, depression, and somatic symptoms, with depression being identified as one of the primary factors contributing to the development of somatic complaints [48]. According to symptom perception theory, bodily cues interact with psychological and environmental factors to produce perceived somatic symptoms, emphasizing the importance of examining these dimensions (negative life events, depression, and somatic symptoms) together [45].

It is worth noting that the multiple mediating model of self-esteem and depression was a chain model rather than a parallel model, which does not show the characteristics of the composite multiple mediating model. Self-esteem and depression are strongly related [49]. Two models tried to explain their nature of relation: the scar model stated that depression erodes self-esteem, while the vulnerability model stated that low self-esteem contributes to depression [50]. A meta-analysis of longitudinal studies provided evidence for supporting the vulnerability model and indicated that the effect of self-esteem on depression was significantly stronger than the effect of depression on self-esteem [50]. This finding can explain why the path of our multiple mediating model is from self-esteem to depression. It has been demonstrated that people with low self-esteem have more negative thoughts and memories when in a negative mood, whereas people with high self-esteem engage more in positive emotional thinking in order to alleviate their negative mood [51]. This indicates that high self-esteem can be a kind of protector with the ability to adequately cope with these feelings, no longer developing into depression [52]. Although there is a significant correlation between self-esteem and somatic symptoms, self-esteem cannot predict somatic symptoms directly. Based on this model, self-esteem shows its modulating effect on depression, and then, its function is transferred by depression to somatic symptoms. Previous studies have pointed out that the accumulation of negative life events can impair an individual’s cognitive function, leading to more depressive symptoms [53]. External social factors may expand the influence through self-cognition, indicating that self-esteem will be affected by negative life events to some extent, and the decrease in self-esteem will increase the individual’s susceptibility to depression. The chain model between self-esteem and depression further proves that the interaction between both has an important effect on the development of somatic symptoms.

## 5. Conclusions

In this study, we observed a high prevalence of somatic symptoms existing in medical students, and we found that different types of negative life events have distinct predictive effects on somatic symptoms, as well as on self-esteem and depression, forming an intermediary variable chain consisting of a multiple mediation model. The results indicate that external social factors, such as negative life events, can affect somatic symptoms through internal psychological factors, such as cognition and mood. Our findings suggest that attention should be paid to the effects of negative life events on the self-esteem and depression status of medical students. Support and help from friends, families, schools, and all sectors of society can help to reduce the incidence and susceptibility of negative life events among medical students. By improving the self-esteem of medical students, it may be possible to further reduce their depression levels, which, in turn, may help to alleviate their somatic symptoms, thereby contributing to their overall physical and mental well-being.

## 6. Limitations

The present study has several limitations that should be acknowledged. Firstly, although the study included more than 3000 participants, all the samples were collected from a single medical university in the north of China. Therefore, the generalizability of the study findings to other populations may be limited, and follow-up research should aim to broaden the scope of investigation. Secondly, this is a cross-sectional study, which only allows for the observation of correlations rather than causation. Therefore, in future research, a longitudinal design should be employed to establish causal relationships. Finally, the study only examined the mediating relationship between several variables and did not conduct a detailed analysis of the influence of potential moderating variables, such as anxiety, coping, and social support. Future research could conduct an in-depth analysis of certain demographic variables that may influence the two mediators of cognitive style and emotion.

## Figures and Tables

**Table 1:** Somatic symptoms of medical students.

Somatic Symptoms Level	Number	Percentage (%)
No somatic symptoms	999	31.03
Mild somatic symptoms	1261	39.17
Moderate somatic symptoms	777	24.14
Severe somatic symptoms	182	5.66

**Table 2:** Individual symptom conditions among medical students with somatic symptoms.

Somatic Symptom	Number	Percentage (%)
1. Stomach pain	1440	64.86
2. Back pain	1306	58.83
3. Pain in arms, legs, or joints (knees, hips, etc.)	1289	58.06
4. Menstrual cramps with periods (females only)	1210	54.50
5. Headaches	1637	73.74
6. Chest pain	899	40.50
7. Nausea, gas, or indigestion	1732	78.02
8. Constipation, loose bowels, or diarrhea	1875	84.46
9. Shortness of breath	672	30.27
10. Dizziness	899	40.50
11. Fainting spells	208	9.37
12. Feeling tired or having low energy	1819	81.94
13. Trouble sleeping	1276	57.48
14. Feeling heart pound or race	1417	63.83
15. Pain or problems during sexual intercourse	172	7.75

**Table 3:** Descriptive statistics and the distribution of somatic symptoms.

Category	Subcategory	Number (%)	Somatic Symptoms ± SD	t/F	*p*
Gender	Male	1399 (43.46%)	5.44 ± 4.15	−19.618	0.000 *
	Female	1820 (56.54%)	8.37 ± 4.52		
Emotional status	Single	2275 (70.67%)	7.13 ± 4.42	0.699	0.485
	Not single	944 (29.33%)	7.01 ± 4.51		
Living expenses	<1000	294 (9.13%)	6.52 ± 4.38	3.020	0.049 *
	1000–2000	2280 (70.83%)	7.12 ± 4.36		
	>2000	645 (20.04%)	7.28 ± 4.76		
One-child	Yes	2068 (64.24%)	6.93 ± 4.50	−2.795	0.005 *
	No	1151 (35.76%)	7.39 ± 4.34		
Religious belief	Yes	129 (4.01%)	7.71 ± 4.36	1.589	0.112
	No	3090 (95.99%)	7.07 ± 4.45		

Note: * *p* < 0.05.

**Table 4:** Correlations between negative life events, self-esteem, depression, and somatic symptoms.

	1	2	3	4
1. Negative life events	1.000			
2. Self-esteem	−0.215 **	1.000		
3. Depression	0.389 **	−0.421 **	1.000	
4. Somatic symptoms	0.314 **	−0.218 **	0.548 **	1.000
Mean	28.350	29.690	3.720	7.100
SD	17.116	4.570	4.298	4.450

Note: ** *p* < 0.01.

**Table 5:** Regression analysis of negative life events and somatization scores.

Negative Life Events	B	SE	β	t	*p*
Punishment	0.194	0.029	0.157	6.596	<0.001
Personal loss	0.231	0.028	0.189	8.225	<0.001
Interpersonal pressure	0.286	0.029	0.256	9.991	<0.001
Learning pressure	0.052	0.026	0.053	1.965	0.05
Health adaptation	−0.263	0.025	−0.271	−10.559	<0.001

**Table 6:** Mediating effects in the mediation model.

Effect	Product of Coefficients		Boot 95% CI	
	Point Estimate	Boot SE	Lower CI	Upper CI
Direct effect: X→Y	0.0310	0.0041	0.0229	0.0391
Indirect effect 1: X→M1→Y	−0.0012	0.0009	−0.0030	0.0006
Indirect effect 2: X→M1→M2→Y	0.0101	0.0012	0.0079	0.0125
Indirect effect 3: X→M2→Y	0.0414	0.0027	0.0363	0.0467
Total effect	0.0813	0.0044	0.0727	0.0898

Note: X: negative life events, M1: self-esteem, M2: depression, and Y: somatic symptoms.

## Data Availability

The data supporting the conclusions of this article will be made available by the authors upon request.
